# Dynamic Loading Regulates Meniscus‐Like Matrix Production in Human Mesenchymal Stromal Cell‐Seeded PET Scaffolds

**DOI:** 10.1002/adhm.71574

**Published:** 2026-08-11

**Authors:** Graciosa Quelhas Teixeira, Luisa de Roy, Anna‐Lotta Feldmeier, Mubashir Ahmad, Sofia Pilão, Maria Ahrens, Anita Ignatius, Carsten Linti, Andreas Martin Seitz

**Affiliations:** ^1^ Institute of Orthopaedic Research and Biomechanics Ulm University Medical Center Ulm Germany; ^2^ German Institutes of Textile and Fiber Research Denkendorf Germany

**Keywords:** dynamic compression, extracellular matrix (ECM) production, mechanobiology, meniscus tissue engineering, mesenchymal stromal cells (MSCs), polyethylene terephthalate (PET) scaffolds

## Abstract

Meniscal injury is a leading cause of early‐onset osteoarthritis, yet regenerative options remain limited. This study investigates a nonwoven polyethylene terephthalate (PET) scaffold for meniscus tissue engineering and assesses its capacity to support mesenchymal stromal cell (MSC) proliferation and chondrogenic differentiation under dynamic loading. Human MSCs are seeded onto PET scaffolds (400–420 g/m^2^, 85% porosity) and cultured for up to 21 days under basal medium (Ctr), chondrogenic differentiation conditions (ChD), or ChD combined with dynamic loading (ChD + Dyn, 12% strain, 1 Hz, 1 h/day, 5 days/week). PET scaffolds support uniform MSC adhesion and colonization. Compared with ChD alone, ChD+Dyn significantly increases cell proliferation and transiently upregulated chondrogenic markers (*SOX9*, *ACAN*, *COL1A1*, *COL2A1*) while suppressing the hypertrophic marker *COL10A1*. Although collagen deposition and construct biomechanics remained unchanged over 21 days, glycosaminoglycan accumulation was reduced in the ChD + Dyn group compared with ChD. RNA sequencing revealed distinct mechanosensitive transcriptional signatures induced by dynamic loading, particularly in genes associated with extracellular matrix remodeling, mechanotransduction, and developmental signaling pathways. These findings demonstrate that nonwoven PET provides a mechanically robust scaffold for meniscus tissue engineering and that dynamic loading promotes MSC proliferation while transiently regulating chondrogenic differentiation and mechanoadaptive matrix remodeling toward a meniscus‐like phenotype.

## Graphical abstract

Dynamic compression enhances mesenchymal stromal cell proliferation in nonwoven PET scaffolds under chondrogenic differentiation conditions and triggers mechanosensitive transcriptional programs associated with extracellular matrix remodeling. These findings highlight the potential of mechanically stimulated PET scaffolds as a promising platform for meniscus tissue engineering. Created in BioRender. Seitz, A. (2026) https://BioRender.com/1yar3mv.

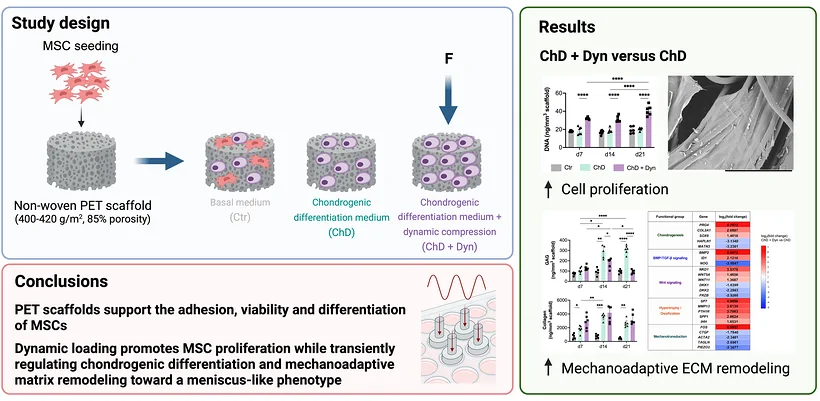

## 1 Introduction

The meniscus is a vital fibrocartilaginous structure in the knee joint, responsible for load distribution, shock absorption, and joint stability [1, 2]. Over 75% of symptomatic osteoarthritis (OA) patients have a history of meniscal injury, highlighting the strong association between meniscal damage and chronic knee pain [3]. Current treatment options range from conservative management to surgery, including meniscectomy, which can relieve symptoms but increases the risk of OA by up to 71% within 20 years [4]. In recent years, meniscal repair has become increasingly preferred over resection, with a 37% rise in repair procedures per surgeon [5, 6]. However, approximately 65% of meniscal tears remain irreparable [7, 8]. In these cases, meniscectomy is often performed [8].

Tissue engineering offers a promising alternative for meniscal repair by generating meniscus‐like constructs using biomaterials, cells, and biomechanical stimulation, thereby addressing the limitations of current surgical treatments, including meniscectomy [2]. A critical factor in this approach is the choice of scaffold material, which must provide both mechanical stability and a biologically supportive environment for cell growth and matrix formation. Most proposed scaffolds for meniscus replacement are either biopolymer‐based, using natural materials such as collagen and hyaluronic acid, or synthetic, composed of polymers such as polylactides and polycaprolactone [9, 10]. Biopolymer scaffolds are known for their biocompatibility and ability to promote tissue regeneration, showing improved outcomes in clinical trials compared to meniscectomy [11, 12, 13]. However, their limited mechanical strength can compromise long‐term joint stability and OA progression [13, 14]. These limitations have motivated the exploration of synthetic alternatives capable of withstanding the demanding mechanical environment of the knee. Synthetic scaffolds offer advantages such as tunable mechanical properties and tailored degradation characteristics, encompassing both nondegradable and controlled biodegradable materials [15, 16]. Among these materials, polyethylene terephthalate (PET) has emerged as a promising candidate for meniscal repair [17] due to its biostability, mechanical durability [18], and biocompatibility [18, 19]. PET is widely used in synthetic fibers and medical devices and has been successfully applied in ligament reconstruction systems, demonstrating satisfactory long‐term outcomes [18, 20, 21, 22, 23]. PET scaffolds provide mechanical support for cell proliferation and can be engineered to enhance extracellular matrix (ECM) formation, facilitating cartilage regeneration and meniscal tissue development [17, 24, 25]. Notably, PET implants have shown long‐term mechanical stability without inducing synovitis or infection in patients undergoing anterior cruciate ligament reconstruction, even after a 10‐year follow‐up [22]. Building on this mechanical reliability, PET represents a promising material for meniscus repair, acting as a supportive matrix for cell proliferation, differentiation, and ECM production.

Mesenchymal stromal cells (MSCs), commonly derived from bone marrow or adipose tissue, are frequently used in meniscal tissue engineering due to their chondrogenic differentiation potential and low immunogenicity [26]. Additionally, MSCs secrete growth factors that reduce inflammation, thereby facilitating meniscal integration and repair [27, 28].

Ultimately, mechanical loading plays a crucial role in meniscus tissue engineering, as it can replicate the physiological environment of the knee joint [2, 29]. Previous studies have demonstrated that dynamic compression enhances meniscus‐specific gene expression, ECM synthesis, and scaffold mechanical properties [30, 31, 32]. Building on this rationale, the present study aimed to evaluate nonwoven PET scaffolds as a biomaterial for meniscus tissue engineering and to determine whether mechanical stimulation regulates human MSC chondrogenic differentiation and meniscus‐like matrix formation. Based on the current literature, we hypothesized that: (i) nonwoven PET supports human MSC adhesion, viability, and differentiation, and is mechanically resilient for meniscus replacement; and (ii) dynamic compression accelerates early chondrogenic differentiation and promotes mechanoadaptive remodeling toward a meniscus‐like ECM. Ultimately, this work explores the interplay between scaffold properties and mechanobiological conditioning, two fundamental determinants of successful meniscus tissue engineering.

## 2 Materials and Methods

### 2.1 Scaffold Fabrication

PET multifilaments were extruded and stretched, resulting in individual filaments with a mean diameter of 17 µm, which were then cut into fibers with approximately 60 mm length and thermally treated in a drying oven to prevent shrinkage during subsequent processing steps. The fibers were processed into a nonwoven fiber web by carding and mechanically consolidated by needle punching. To obtain the desired porosity, the resulting needle felt was passed through a controlled gap between two heated calendar rolls, where the fleece thickness was thermally set. This process produced nonwoven scaffolds with a thickness of approximately 1.8 mm, weight of 400–420 g/m^2^, and 85% porosity [33].

### 2.2 Mesenchymal Stromal Cell Expansion and Culture on PET Scaffolds

Adipose‐derived MSCs from a single donor (male, 38 years; Lonza, Basel, Switzerland) were expanded in basal MSC medium (Lonza) to passages 6–9. Experiments included 1–2 replicates per passage, with all groups within each experiment using MSCs from the same passage. The experimental timeline is outlined in Figure 1A.

**FIGURE 1:**
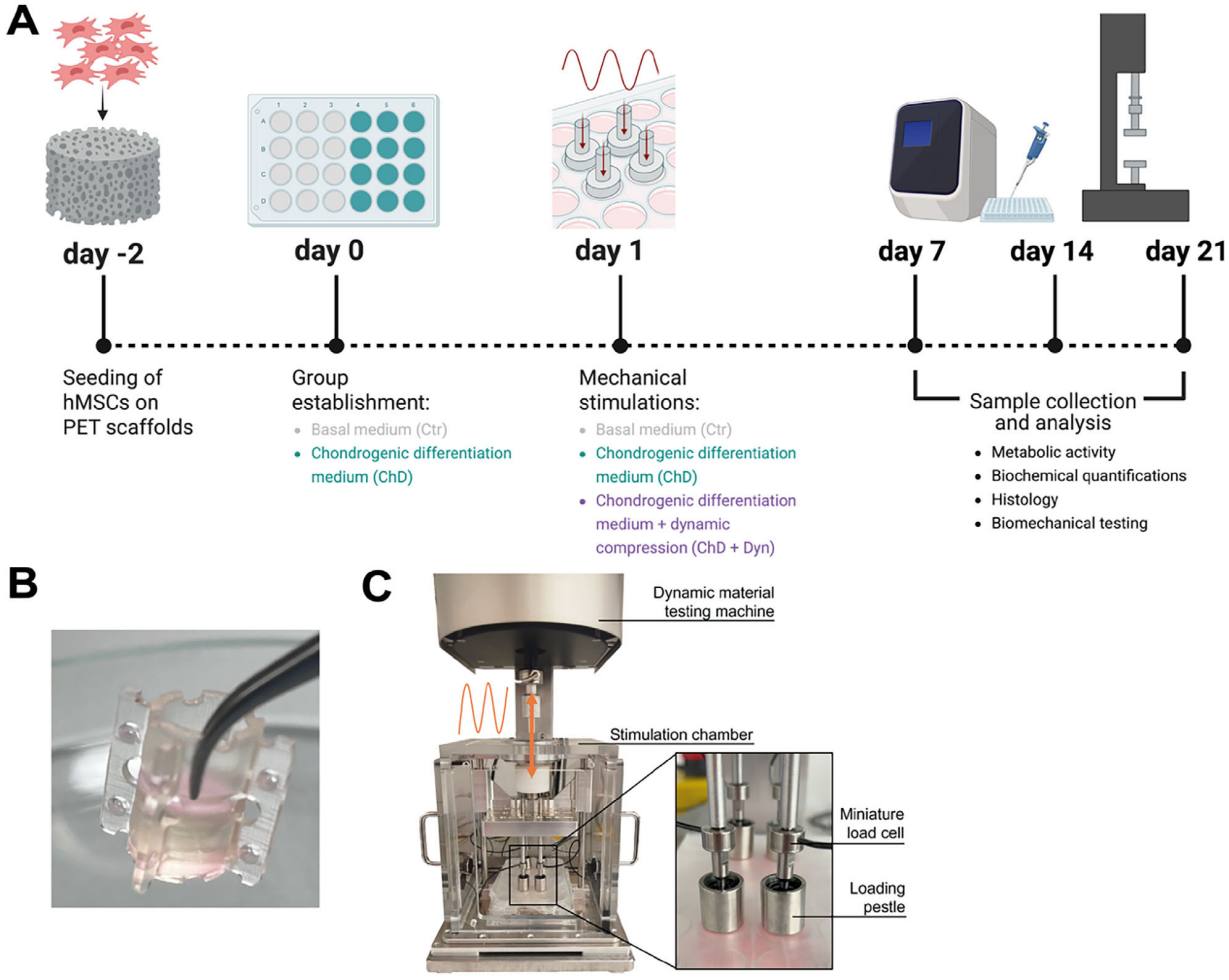
Experimental groups and workflow. (A) Experimental timeline. PET scaffolds were seeded with human mesenchymal stromal cells (hMSCs). The experiment included three groups: control (Ctr), chondrogenic differentiation (ChD), and ChD combined with dynamic loading (ChD + Dyn). The Ctr group was maintained in standard hMSC medium under normoxic conditions (21% O_2_ and 5% CO_2_), while both the ChD and ChD + Dyn groups were cultured in ChD medium under hypoxic conditions (5% O_2_ and 8.5% CO_2_). The ChD + Dyn group underwent dynamic loading at a frequency of 1 Hz and 12% strain for 1 h/day, 5 days/week. Samples were collected for analysis at days 7, 14, and 21. Created in BioRender. Seitz, A. (2026). https://BioRender.com/38rhxt6 (B) Custom‐made 3D‐printed holder for a PET scaffold. (C) Material testing machine equipped with a custom‐made stimulation chamber, which enabled a 24‐well plate to be placed under sterile conditions. Custom 3D‐printed pestles were positioned at the top and bottom of each scaffold to transfer the load to individual samples.

PET scaffolds (7 mm diameter × 2 mm thickness) were placed in custom 3D‐printed holders (Figure 1B) in 24‐well plates and seeded with 2.2 × 10^5^ MSCs per scaffold. To improve cell distribution, the scaffolds were inverted after 1 h and returned to their original orientation after an additional 3 h. Following 2 days of culture under basal conditions (37°C, humidified atmosphere), constructs were randomly assigned to three groups: static control group (Ctr; basal MSC medium, 21% O_2_ and 5% CO_2_), chondrogenic differentiation conditions (ChD) combining chondrogenic differentiation medium (Lonza) supplemented with 10 ng/mL recombinant human transforming growth factor (TGF)‐β3 (R&D Systems, Minneapolis, MN, USA) and hypoxia (5% O_2_ and 8.5% CO_2_), or ChD with dynamic loading (ChD + Dyn). Dynamic loading was applied as sinusoidal axial compression (12% strain, 1 Hz, 1 h/day, 5 days/week) [34, 35, 36] using a material testing machine (ElectroForce 5500, Bose Corporation – ElectroForce Systems Group, Eden Prairie, MN, USA) equipped with a custom sterile loading chamber and 3D‐printed pestles positioned at the top and bottom of each scaffold for individual load transfer (Figure 1C). Constructs and supernatants were analyzed after 7, 14, and 21 days of culture, as reported for similar studies [37, 38]. RNA sequencing and inflammatory protein array analyses were performed only on Day 21.

### 2.3 MSC Viability and Metabolic Activity

#### 2.3.1 Mitochondrial Metabolic Activity

Cell metabolic activity was assessed by resazurin reduction assay [39]. Constructs were incubated with 0.02 mg/mL resazurin sodium salt (Sigma–Aldrich, St. Louis, MO, USA) in basal medium for 3 h at 37°C. Fluorescence was measured using a spectrophotometer (Spark, Tecan, Männedorf, Switzerland) at 530 nm excitation and 590 nm emission.

#### 2.3.2 DNA Quantification in the MSC‐Seeded PET Constructs and Culture Supernatants

Before DNA quantification, cells were lysed in proteinase K solution (0.5 mg/mL in 30 mM Tris‐HCl, pH 8.0; Sigma–Aldrich) for 1 h at 37°C followed by 48 h at 56°C with shaking. DNA content quantified with the Quant‐iT PicoGreen dsDNA Assay Kit (Invitrogen, Carlsbad, CA, USA) according to the manufacturer's instructions.

#### 2.3.3 Glucose and Lactate Quantification in Culture Supernatants

Culture supernatants were heat‐inactivated (80°C, 15 min) and centrifuged at 5,000 × g for 5 min. Glucose and L‐lactate concentrations were quantified using colorimetric assay kits (Sigma–Aldrich) following the manufacturer's instructions. Glucose consumption was determined by subtracting the glucose concentration in conditioned medium from that in fresh medium.

### 2.4 Chondrogenic Gene Expression Analysis

Gene expression was assessed by quantitative PCR (qPCR). Total RNA was extracted from fragmented MSC‐seeded PET constructs using QIAzol (Qiagen, Düsseldorf, Germany) followed by phase separation with 200 µL chloroform and centrifugation at 12,000 × g for 15 min at 4°C. The aqueous phase was mixed with an equal volume of 70% ethanol, and RNA was isolated using the PureLink RNA Mini Kit (Thermo Fisher Scientific, Waltham, MA, USA) with on‐column DNase treatment (Qiagen). RNA was eluted in RNase‐free water and quantified using a NanoQuant spectrometer (Spark, Tecan Group Ltd., Männedorf, Switzerland). For cDNA synthesis with integrated removal of DNA contamination, 1 µg of RNA was treated with a QuantiTect reverse transcription kit (ThermoFisher Scientific). qPCR was performed on a QuantStudio 3 system (Applied Biosystems, Waltham, MA, USA) using Platinum SYBR Green SuperMix‐UDG with ROX (Invitrogen, Waltham, MA, USA) for the reference gene human glyceraldehyde 3‐phosphate dehydrogenase (*GAPDH*) and target genes listed in Table S1. Melting curve analysis confirmed amplification specificity, and reactions with Cq > 35 were excluded. Relative expression was calculated using the 2^−ΔΔCt^ method [40].

### 2.5 Extracellular Matrix Production

#### 2.5.1 Scanning Electron Microscopy

Cell‐seeded PET constructs were fixed with glutaraldehyde, treated with osmium tetroxide, air‐dried, and imaged in the transverse plane using a benchtop scanning electron microscope (TM‐1000, Hitachi, Tokyo, Japan).

#### 2.5.2 GAG and Collagen Quantification

GAG and collagen contents were quantified from the digests obtained for DNA quantification (Section 2.3.2). GAG content was determined using the 1,9–dimethylmethylene blue assay with chondroitin sulfate (Sigma–Aldrich) as the standard [41, 42]. Hydroxyproline was quantified for collagen extrapolation, as previously described [43]. Collagen content was estimated by assuming that hydroxyproline represents approximately 10% of the total collagen mass [44, 45].

### 2.6 Inflammation and Matrix Degradation

#### 2.6.1 Inflammation Mediator Analysis

Prostaglandin E_2_ (PGE_2_) was quantified in culture supernatants by enzyme‐linked immunosorbent assay (Arbor Assays, Ann Arbor, MI, USA) following the manufacturer's instructions. Inflammation‐associated proteins were profiled at Day 21 using the MILLIPLEX Human Cytokine/Chemokine/Growth Factor Panel A (HCYTA‐60K, Merck Millipore, Burlington, MA, USA) according to the manufacturer's protocol. A total of 48 inflammatory mediators were measured using the Luminex 100 Total System (Bio‐Rad Laboratories, Hercules, CA, USA).

#### 2.6.2 Zymography

The activity of MMPs 2 and 9 was assessed in culture supernatants by gelatine zymography, as previously described [46]. Briefly, 15–20 µg of total protein, determined using the Pierce BCA Protein Assay (Thermo Fisher Scientific), was separated on 10% polyacrylamide gels containing 0.1% gelatine. Following electrophoresis, gels were washed in 2% Triton X‐100 and incubated for 16 h at 37°C in 50 mM Tris‐HCl, pH 7.5, with 10 mM CaCl_2_. Gels were stained with 0.1% Coomassie Brilliant Blue R‐250 in 50% methanol/10% acetic acid, and MMP activity was quantified by densitometry.

### 2.7 RNA Sequencing

RNA samples with an RNA integrity number > 9.5 were used for RNA sequencing by Novogene (Cambridge, UK) as previously described [47]. Poly(A)‐enriched libraries were sequenced on an Illumina platform. Raw sequencing reads were quality filtered by removing adapter‐contaminated reads, reads containing > 10% ambiguous nucleotides, and reads with > 50% low‐quality bases, generating clean reads for downstream analyses. Clean reads were aligned to the human reference genome (GRCh38/hg38) using hierarchical indexing for Spliced Alignment of Transcripts 2. Gene‐level read counts were obtained for 58 735 annotated genes. Differential expression analysis was performed using the DESeq2 package, which normalizes raw read counts to account for differences in sequencing depth while retaining all quantified genes for statistical analysis. Statistical significance was assessed using the Wald test with Benjamini–Hochberg correction for multiple testing. Genes with |log_2_(fold change)| ≥ 1.0 and an adjusted *p‐*value ≤ 0.05 were considered differentially expressed genes (DEGs). Downstream analyses were performed using the NovoMagic Plus platform (Novogene). Sample repeatability and variability were evaluated by principal component analysis (PCA) and Pearson correlation heatmap analysis. Differential gene expression was visualized using volcano plots. Gene Ontology (GO) enrichment analysis was performed using significantly DEGs (|log_2_FC| ≥ 1 and adjusted *p* ≤ 0.05) with the clusterProfiler package [48]. Enriched GO terms were ranked according to their adjusted *p* values, and the 10 most significantly enriched terms from each of the three GO domains (biological process, cellular component, and molecular function) were selected for visualization.

### 2.8 Biomechanical Characterization of MSC‐Seeded PET Constructs

Confined compression relaxation tests were conducted to determine the permeability (k) and equilibrium modulus (Eeq) of PET constructs after culture, following an established protocol [49, 50]. Briefly, cylindrical samples (5 mm diameter) were punched from each construct, and height was measured with a digital caliper. Samples were placed in a confined compression chamber with an impermeable base and a porous Al_2_O_3_ plate on top to permit fluid flow, submerged in PBS during testing. A 20 N load cell (Z010, Zwick Roell, Ulm, Germany) applied three sequential strain levels (*εi* = 0.1, 0.15, and 0.2), each held for 15 min to reach equilibrium. Permeability and equilibrium modulus were calculated by fitting the Mow et al. [11] diffusion equation using nonlinear least squares.

### 2.9 Statistical Analysis

A priori power analysis was conducted to determine the sample size required to detect a biologically relevant effect of MSC differentiation in PET scaffolds. Based on previous studies of MSC chondrogenic differentiation and GAG production on Day 21 [38], we assumed a moderate effect size (Cohen's d) = 2.5. Using a two‐tailed one‐sample *t*‐test with *α* = 0.05 and desired power of 0.8, the minimum sample size was calculated as *n* = 4 per group using G*Power software (version 3.1.9.6). This sample size was deemed sufficient to detect meaningful differences in differentiation outcomes. However, for RNA sequencing, we used *n* = 3 as the sample size emphasizing an explorative character of these results. Statistical analysis, excluding RNA sequencing, was performed using GraphPad Prism 11 (GraphPad software, Inc, La Jolla, CA, USA), except for RNA sequencing data (Section 2.7). Groups were tested for normal distribution using the Shapiro–Wilk test. Nonparametric data were analyzed by Kruskal–Wallis test with Dunn's multiple comparison test was used. Two‐way analysis of variance (ANOVA) was used for normally distributed data. This allowed comparison between ChD and Ctr, and ChD + Dyn and ChD groups. Significant differences were considered at *p* < 0.05.

## 3 Results

### 3.1 MSC Viability and Metabolic Activity

Mitochondrial metabolic activity of MSCs was detectable across all conditions (Figure 2A), confirming sustained cell viability throughout the study. The ChD group exhibited significantly lower metabolic activity than the Ctr at Day 21 (*p* < 0.05) and the ChD + Dyn at all time points (*p* < 0.05). DNA content was also lower in ChD versus ChD + Dyn at all time points (*p* < 0.05, Figure 2B). In the ChD + Dyn group, metabolic activity and DNA significantly increased from Day 7 to 21 (*p* < 0.05). However, when mitochondrial activity was normalized to DNA content (Figure 2C), no significant differences were observed between groups, indicating that the increase in metabolic activity was primarily attributable to increased cell number rather than enhanced metabolic activity on a per‐cell basis. DNA levels in the supernatant were consistently lower in ChD relative to the Ctr at Days 7 and 21 (*p* < 0.001, Figure 2D), and relative to the ChD + Dyn group at Days 7, 14, and 21 (*p* < 0.05). While DNA release in ChD + Dyn group remained stable over time, ChD showed progressive accumulation, with significantly higher values at Day 21 compared to earlier time points (*p* < 0.01). Moreover, ChD displayed significantly higher glucose consumption and lactate production than the Ctr group at all time points (*p* < 0.01, Figure 2E–F). Lactate production was also higher in ChD than ChD + Dyn across all time points (*p* < 0.01) and increased significantly at Day 21 relative to Days 7 and 14 (*p* < 0.01).

**FIGURE 2:**
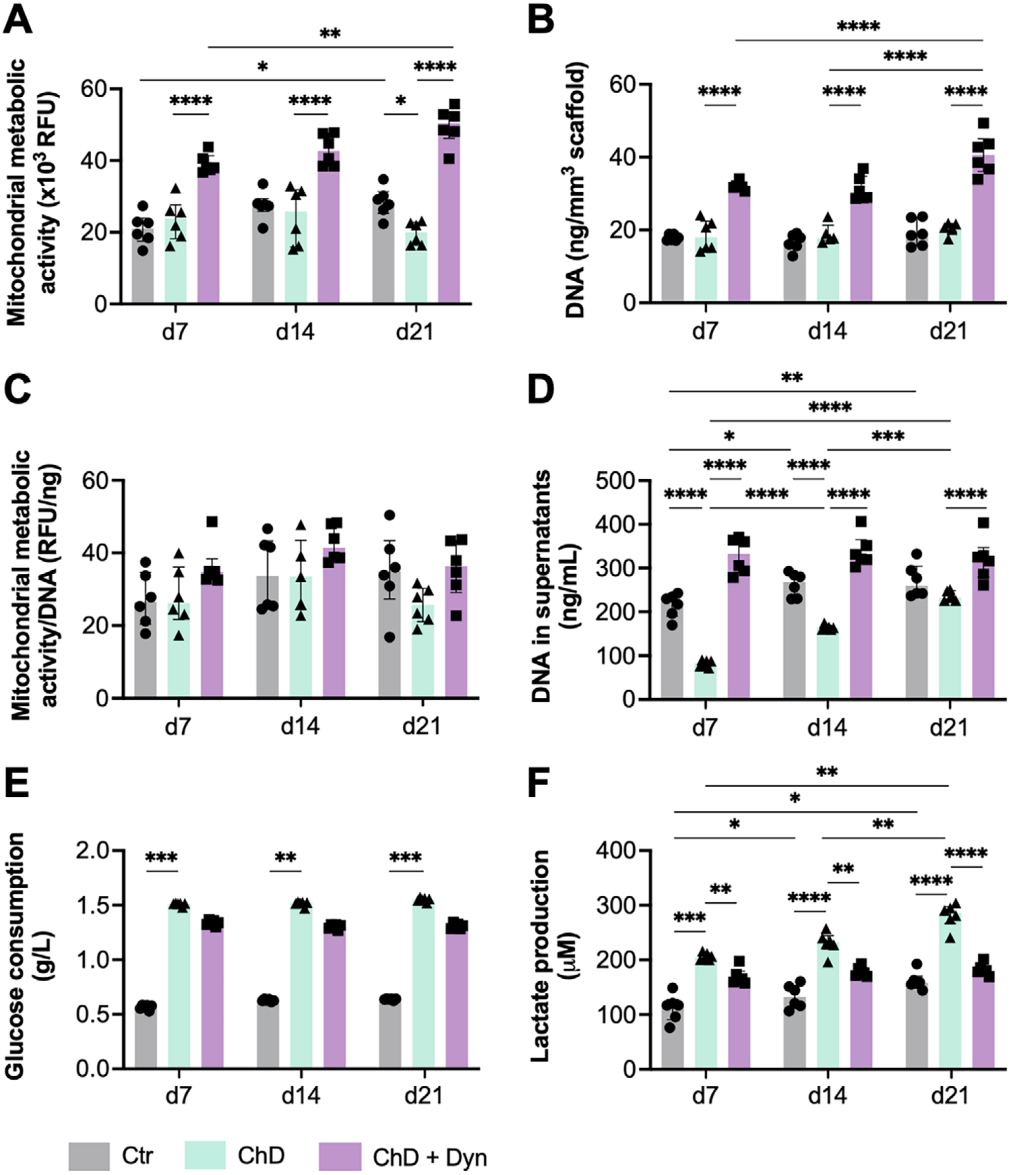
MSC viability and metabolic activity at Days 7, 14, and 21 of culture under control conditions (Ctr), chondrogenic differentiation (ChD), and ChD combined with dynamic loading (ChD + Dyn). (A) Mitochondrial metabolic activity in relative fluorescence units (× 10^3^ RFU). (B) DNA (ng/mm^3^ scaffold). (C) Mitochondrial metabolic activity normalized to DNA (RFU/ng). (D) DNA in culture supernatants (µg/mL). (E) Glucose consumption (g/L). (F) Lactate production (µM). Data are presented as bar plots and as median with interquartile range (*n* = 1 MSC donor; 5–6 independent experimental replicates). * *p* < 0.05, ** *p* < 0.01, *** *p* < 0.001, and **** *p* < 0.0001.

### 3.2 Chondrogenic Gene Expression Analysis

MSCs cultured on PET scaffolds under different conditions exhibited significant temporal and treatment‐dependent differences in the expression of chondrogenic markers (Figure 3). Aggrecan (*ACAN*) expression was significantly lower in the ChD group than in the Ctr across all time points (*p* < 0.05, Figure 3A). On Day 7, ChD + Dyn further downregulated the expression of *ACAN* (*p* < 0.05), but this pattern reversed at Day 14 (*p* < 0.001). By Day 21, *ACAN* levels converged between the ChD and ChD + Dyn groups. SRY‐box transcription factor 9 (*SOX9*), an early chondrogenic transcription factor, was significantly downregulated in ChD relative to Ctr (*p* < 0.05, Figure 3B) and ChD + Dyn (*p* < 0.01) groups at Days 14 and 21. Notably, only ChD + Dyn induced a significant increase in *ACAN* and *SOX9* expression over time, peaking at Day 14, indicating enhanced chondrogenic activation under dynamic loading. Collagen type I alpha 1 chain (*COL1A1*) expression was upregulated in ChD compared to Ctr at all time points (*p* < 0.05, Figure 3C). It remained relatively stable under ChD conditions but showed a transient peak in the ChD + Dyn group at Day 14. A similar pattern was observed for *COL2A1* (Figure 3D), which peaked at Day 14 in ChD + Dyn. In contrast to *COL1A1*, *COL2A1* remained upregulated at Day 21 compared with ChD (*p* < 0.05). Expression of the hypertrophy‐associated marker *COL10A1* increased over time in the ChD group compared with the Ctr (*p* < 0.05, Figure 3E), but this effect was suppressed in the ChD + Dyn group at Day 14 (*p* < 0.01).

**FIGURE 3:**
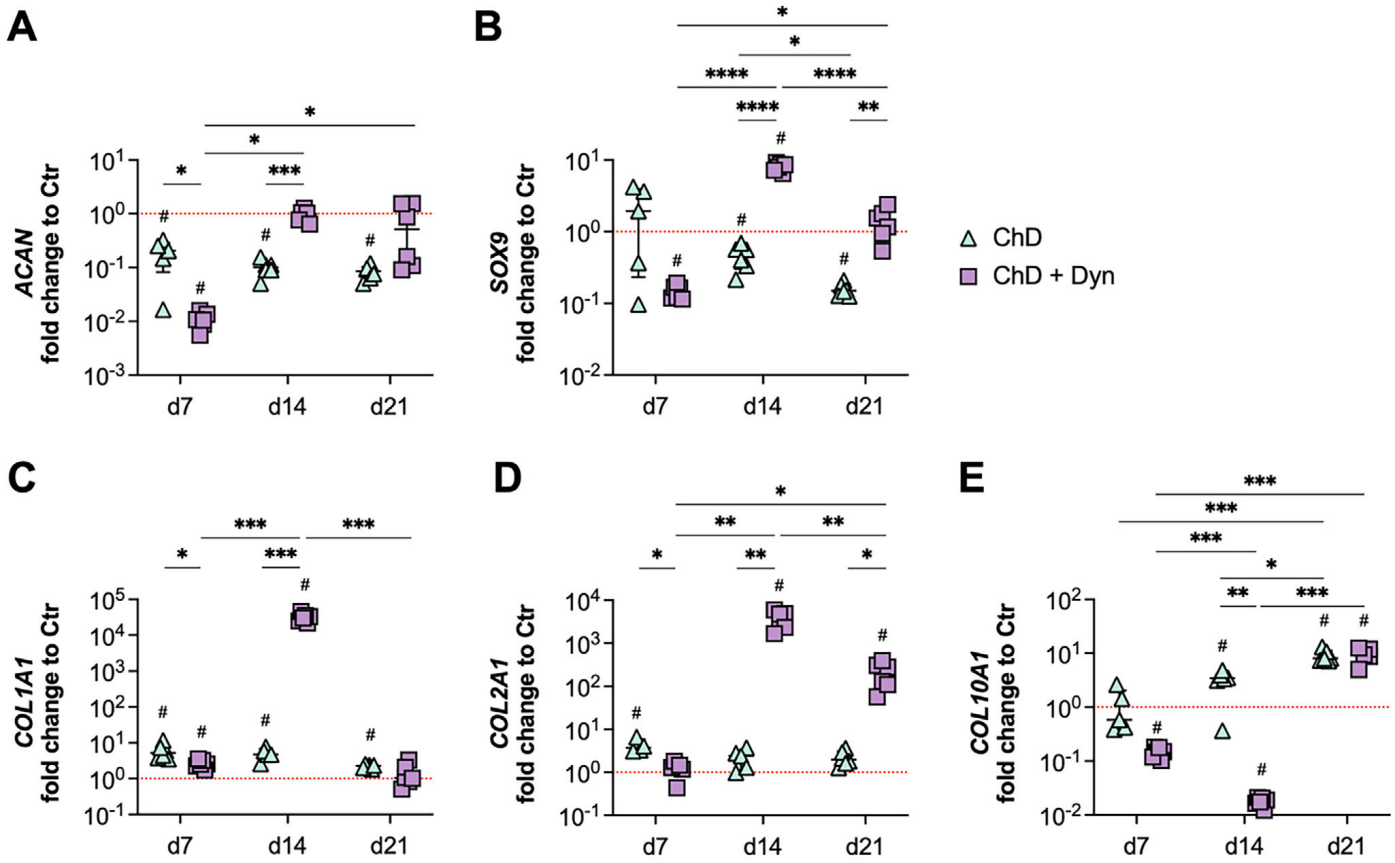
Relative gene expression of chondrogenic differentiation markers. (A) mRNA fold change of *ACAN*, (B) *SOX9*, (C) *COL1A1*, (D) *COL2A1*, and (E) *COL10A1* in MSCs cultured for 7, 14, and 21 days in PET scaffolds under basal conditions (Ctr), chondrogenic differentiation medium (ChD), and ChD combined with dynamic loading (ChD + Dyn). Levels of mRNA were normalized to *GAPDH* and to Ctr group (red dashed line = 1). Results are presented as 2^−ΔΔCt^. Data are presented as dot plots and as median with interquartile range (*n* = 1 MSC donor; 4–6 independent experimental replicates). ^#^ *p* < 0.05 (^#^, comparison between Ctr and ChD); * *p* < 0.05, ** *p* < 0.01, *** *p* < 0.001, **** *p* < 0.0001 (*, comparison between ChD and ChD + Dyn).

### 3.3 Extracellular Matrix Production

Representative SEM images acquired on Day 1 demonstrated the presence of MSCs on the upper surface, central region, and lower surface of the PET scaffolds, indicating successful cell colonization (Figure S1). ECM formation was subsequently observed by SEM in the central region of the constructs (Figure 4A). Representative SEM images from the upper, central, and lower scaffold regions (Figure S2), together with whole cross‐sectional Safranin O/Fast Green staining (Figure S3) confirmed ECM deposition throughout the scaffolds. In the Ctr group, sparse and disorganized fibrillar structures persisted throughout the culture period, indicating minimal ECM deposition. In contrast, both the ChD and ChD + Dyn groups exhibited progressive ECM accumulation by Days 14 and 21. Biochemical analysis showed that GAG content increased from Day 7 to 14 in the ChD group (*p* < 0.05, Figure 4B) and was significantly greater than in the Ctr and ChD + Dyn groups at Days 14 and 21 (*p* < 0.05). Collagen content increased from Day 7 to 14 in the ChD group (*p* < 0.01, Figure 4C) and was higher than in the Ctr group (*p* < 0.05). No differences in collagen content were observed between the ChD and ChD + Dyn groups at any time point. Normalization to DNA revealed that both GAG/DNA and collagen/DNA were significantly lower in the ChD + Dyn group than in the ChD group at Days 14 and 21 (*p* < 0.01; Figure 4D–E). GAG release into the supernatant was transiently increased in ChD + Dyn at Day 7 (*p* < 0.05, Figure S4A), whereas collagen release remained low across all conditions, increasing slightly in ChD by Day 21 (*p* < 0.05, Figure S4B).

**FIGURE 4:**
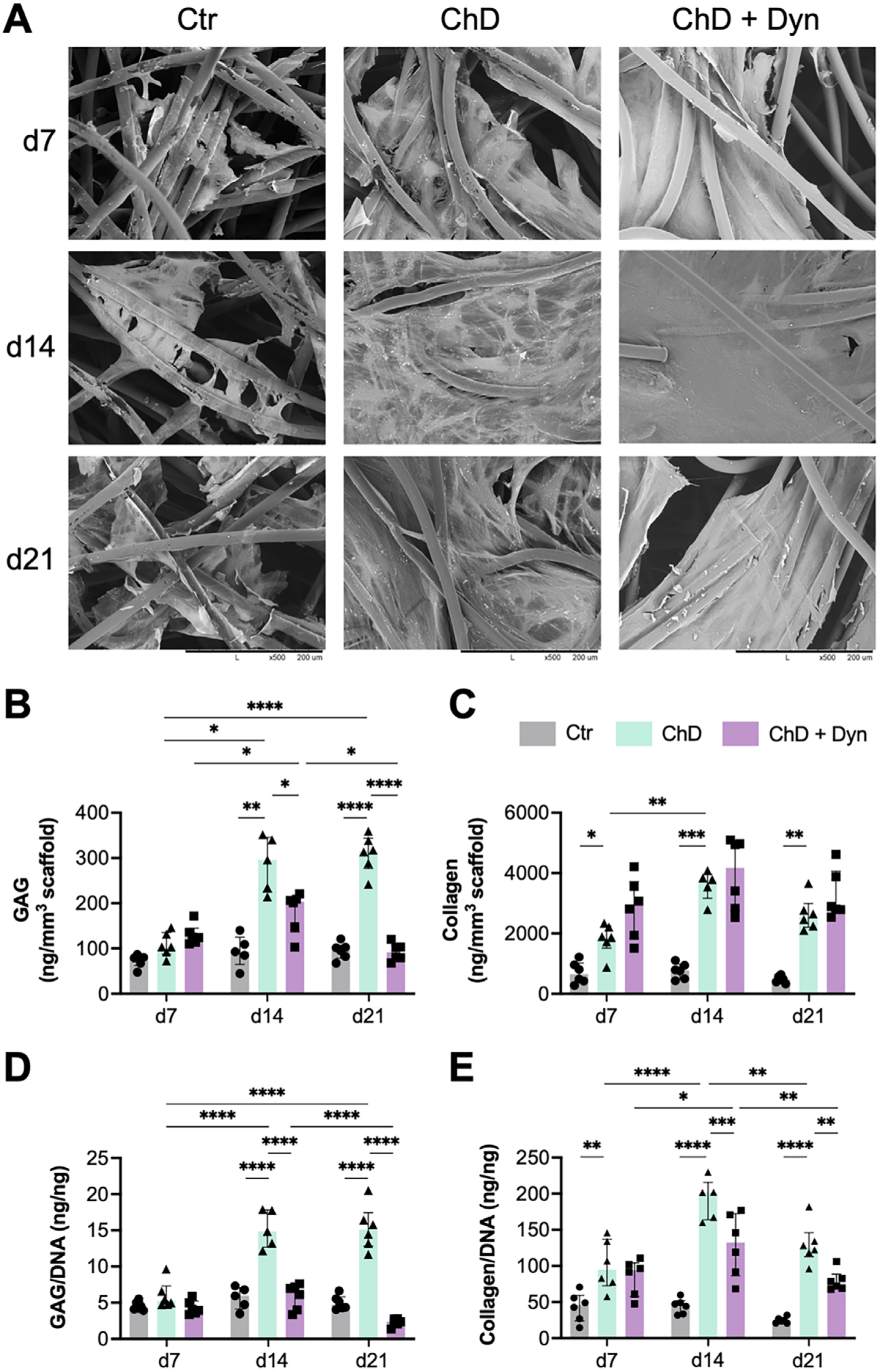
Extracellular matrix production by MSCs seeded on PET scaffolds at Days 7, 14, and 21 of culture. (A) Representative scanning electron microscopy images of the central region of MSC‐seeded PET constructs cultured for 7, 14, and 21 days under basal conditions (Ctr), chondrogenic differentiation medium (ChD), and ChD combined with dynamic loading (ChD + Dyn). Scale bar, 200 µm. (B) GAG (ng/mm^3^ scaffold). (C) Collagen (ng/mm^3^ scaffold). (D) GAG normalized to DNA (ng/ng). (E) Collagen normalized to DNA (ng/ng). Data are presented as bar plots and as median with interquartile range (*n* = 1 MSC donor; 5–6 independent experimental replicates). * *p* < 0.05, ** *p* < 0.01, *** *p* < 0.001, **** *p* < 0.0001.

### 3.4 Inflammation and Matrix Degradation

To assess the mechanosensitivity, PGE_2_ production was measured (Figure 5A). Across all time points, PGE_2_ levels were unchanged by ChD relative to Ctr; however, ChD + Dyn significantly increased PGE_2_ production compared with ChD alone at days 7 and 14 (*p* < 0.01), confirming active cellular response to dynamic compression. Metalloproteinase (MMP) activity, key to ECM remodeling, was analyzed by gelatine zymography (Figure 5B). MMP‐2 was consistently detected, though only in its inactive (pro‐) form (Figure 5C). Significantly more pro‐MMP‐2 production was observed in ChD compared with Ctr and ChD + Dyn at Days 7 and 21 (*p* < 0.05). Pro‐MMP‐9 activity decreased from Day 7 to 14 and remained low at Day 21 in the Ctr group (*p* < 0.01; Figure 5D). In contrast, pro‐MMP‐9 activity was not detected in the ChD and ChD + Dyn groups at Day 7 but increased under ChD + Dyn conditions between Days 14 and 21 (*p* < 0.01), and by Day 21, levels were significantly higher than in the ChD group (*p* < 0.01). The active MMP‐9 form was only sporadically detected (Figure 5E).

**FIGURE 5:**
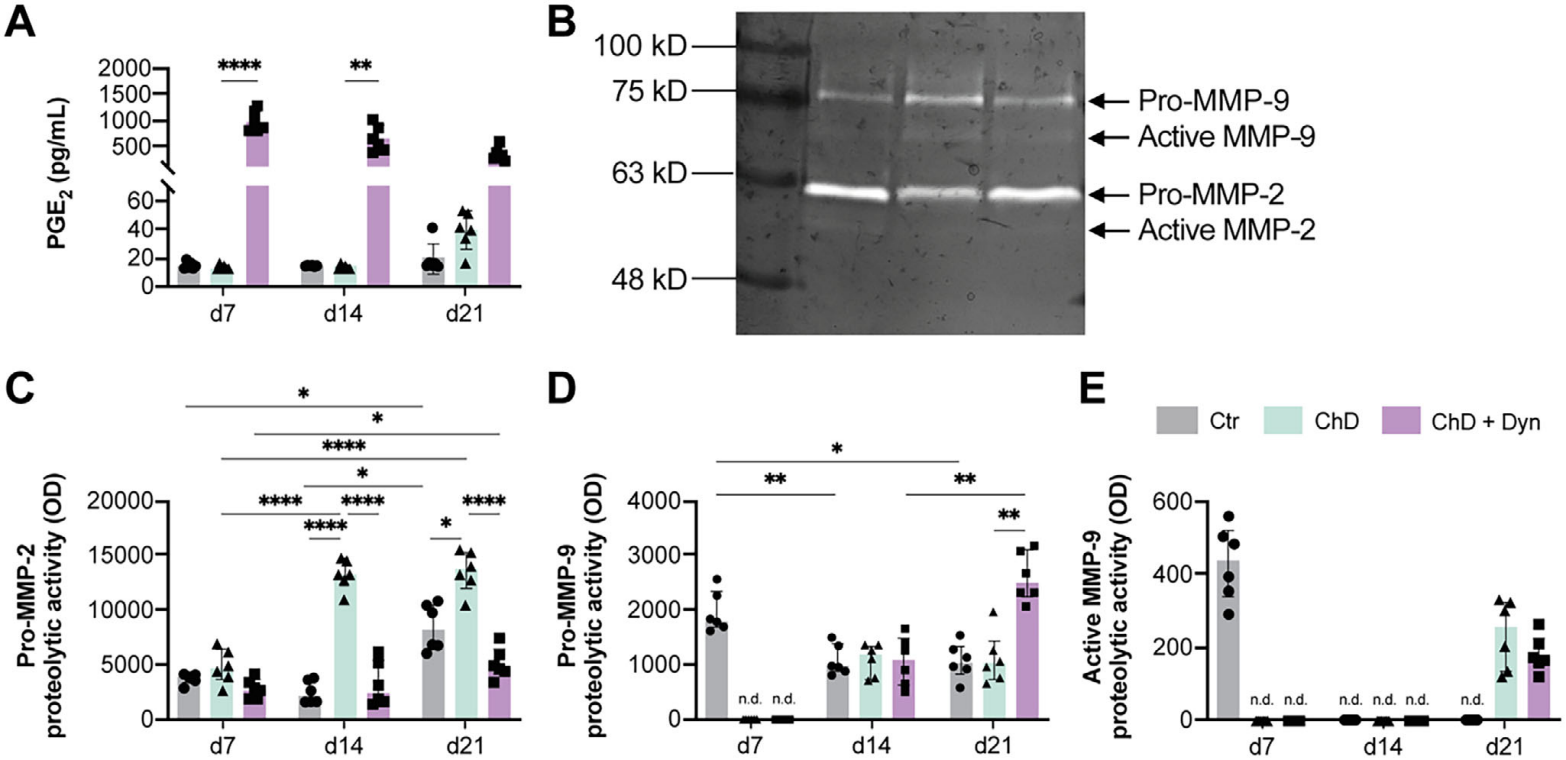
Protein release by MSCs seeded on PET scaffolds to the culture supernatants at Days 7, 14, and 21 of culture under control conditions (Ctr), chondrogenic differentiation (ChD), and ChD combined with dynamic loading (ChD + Dyn). (A) PGE_2_ (pg/mL). (B) Representation gelatine zymography image for determination of MMP activity. (C) Pro‐MMP‐2, (D) pro‐MMP‐9, and (E) active MMP‐9 proteolytic activity (OD). Data are presented as bar plots and as median with interquartile range (*n* = 1 MSC donor; 6 independent experimental replicates). * *p* < 0.05, ** *p* < 0.01, **** *p* < 0.0001.

The proteins detected by the inflammatory mediators panel are presented in Figure 6. ChD markedly altered the inflammatory secretome of MSCs compared with the control group (Ctr). Within the panel of pro‐inflammatory cytokines (Figure 6A), ChD increased the secretion of interferon gamma (IFNγ; *p* < 0.05), while reducing interleukin (IL)‐7 (*p* < 0.001), IL‐27 (*p* < 0.01), tumor necrosis factor beta (TNFβ; *p* < 0.01), and granulocyte‐macrophage colony‐stimulating factor (GM‐CSF; *p* < 0.0001). The addition of dynamic loading decreased IL‐6 (*p* < 0.01), IL‐12(p40) (*p* < 0.05), and IL‐17A (*p* < 0.001), while increasing GM‐CSF (*p* < 0.05). Among the regulatory cytokines (Figure 6B), ChD increased the secretion of IL‐4 (*p* < 0.0001) and IL‐13 (*p* < 0.001), whereas IL‐3 and IL‐9 remained unchanged. Compared to ChD, ChD + Dyn increased the production of IL‐3 (*p* < 0.05) and attenuated the ChD‐induced increase in IL‐4 (*p* < 0.0001) and IL‐13 (*p* < 0.001). IL‐9 remained unaffected across all experimental groups. The chemokine profile (Figure 6C) was characterized by increased secretion of eotaxin (*p* < 0.001), growth‐regulated oncogene alpha (GROα; *p* < 0.0001), and IL‐8 (*p* < 0.0001), together with reduced interferon gamma‐induced protein 10 (IP‐10; *p* < 0.0001), monocyte chemoattractant protein (MCP)‐1 (*p* < 0.0001), MCP‐3 (*p* < 0.0001), and regulated upon activation, normal T‐cell expressed and secreted (RANTES; *p* < 0.05). Macrophage inflammatory protein‐1 alpha (MIP‐1α) was unaffected. ChD + Dyn selectively reduced eotaxin (*p* < 0.05), GROα (*p* < 0.01), and IL‐8 (*p* < 0.0001), without affecting the remaining chemokines. Within the growth factor group (Figure 6D), ChD decreased macrophage colony‐stimulating factor (M‐CSF; *p* < 0.0001) while increasing platelet‐derived growth factor‐AA (PDGF‐AA; *p* < 0.05) and vascular endothelial growth factor A (VEGF‐A; *p* < 0.0001). Fibroblast growth factor‐2 (FGF‐2) and Fms‐like tyrosine kinase 3 ligand (FLT‐3L) remained unchanged. ChD + Dyn increased M‐CSF levels (*p* < 0.01) and reduced VEGF‐A secretion (*p* < 0.0001) compared with ChD alone. The remaining growth factors were unaffected.

**FIGURE 6:**
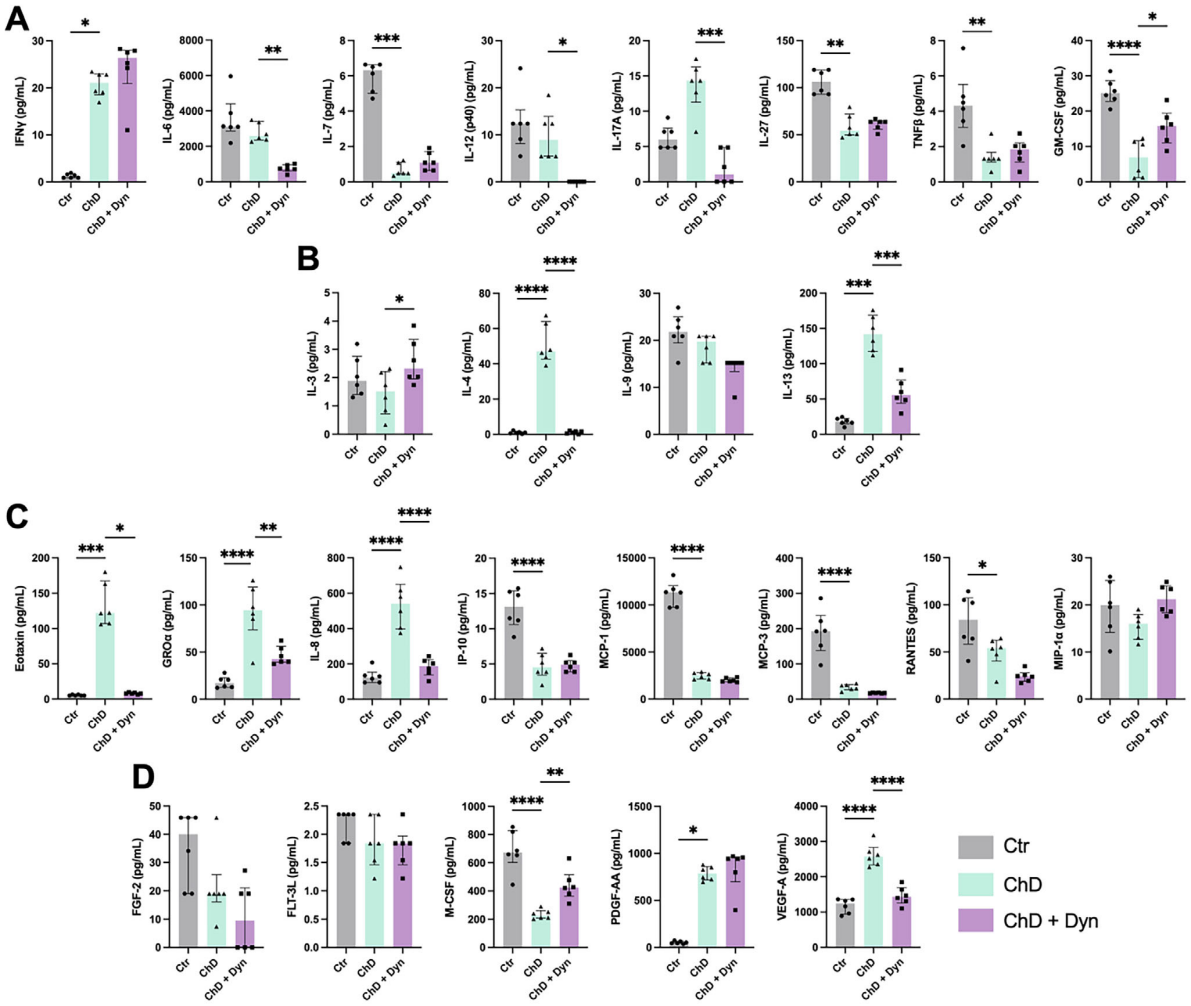
Inflammatory response of MSCs seeded on PET scaffolds after 21 days of culture under control conditions (Ctr), chondrogenic differentiation (ChD), and ChD combined with dynamic loading (ChD + Dyn). Multiplex analysis of inflammatory mediators released into the culture supernatants. Analytes are grouped according to their biological function. (A) Pro‐inflammatory cytokines: interferon gamma (IFNγ), interleukin (IL)‐6, IL‐7, IL‐12(p40), IL‐17A, IL‐27, tumor necrosis factor beta (TNFβ), and granulocyte‐macrophage colony‐stimulating factor (GM‐CSF). (B) Regulatory cytokines: IL‐3, IL‐4, IL‐9, and IL‐13; (C) chemokines: eotaxin (CCL11), growth‐regulated oncogene alpha (GROα/CXCL1), IL‐8 (CXCL8), interferon gamma‐induced protein 10 (IP‐10/CXCL10), monocyte chemoattractant protein‐1 (MCP‐1/CCL2), monocyte chemoattractant protein‐3 (MCP‐3/CCL7), regulated upon activation, normal T‐cell expressed and secreted (RANTES/CCL5), and macrophage inflammatory protein‐1 alpha (MIP‐1α/CCL3). (D) Growth factors: fibroblast growth factor‐2 (FGF‐2), Fms‐like tyrosine kinase 3 ligand (FLT‐3L), macrophage colony‐stimulating factor (M‐CSF), platelet‐derived growth factor‐AA (PDGF‐AA), and vascular endothelial growth factor A (VEGF‐A). Data are presented as bar plots and as median with interquartile range (n = 1 MSC donor; 6 independent experimental replicates). * *p* < 0.05, ** *p* < 0.01, *** *p* < 0.001, **** *p* < 0.0001.

### 3.5 RNA Sequencing

PCA showed clear separation between Ctr, ChD, and ChD+Dyn groups, indicating distinct treatment‐specific transcriptional differences (Figure 7A). Consistently, Pearson correlation analysis demonstrated high intra‐group similarity among biological replicates (Figure 7B). The transcriptomic comparison between ChD and Ctr is presented in Figure S5. The lists of DEGs and the corresponding GO enrichment analyses for the ChD versus Ctr and ChD + Dyn versus ChD comparisons are provided in Supporting Data 2–5, with the DEG lists in Supporting Data 2 and 3 and the GO enrichment analyses in Supporting Data 4 and 5.

**FIGURE 7:**
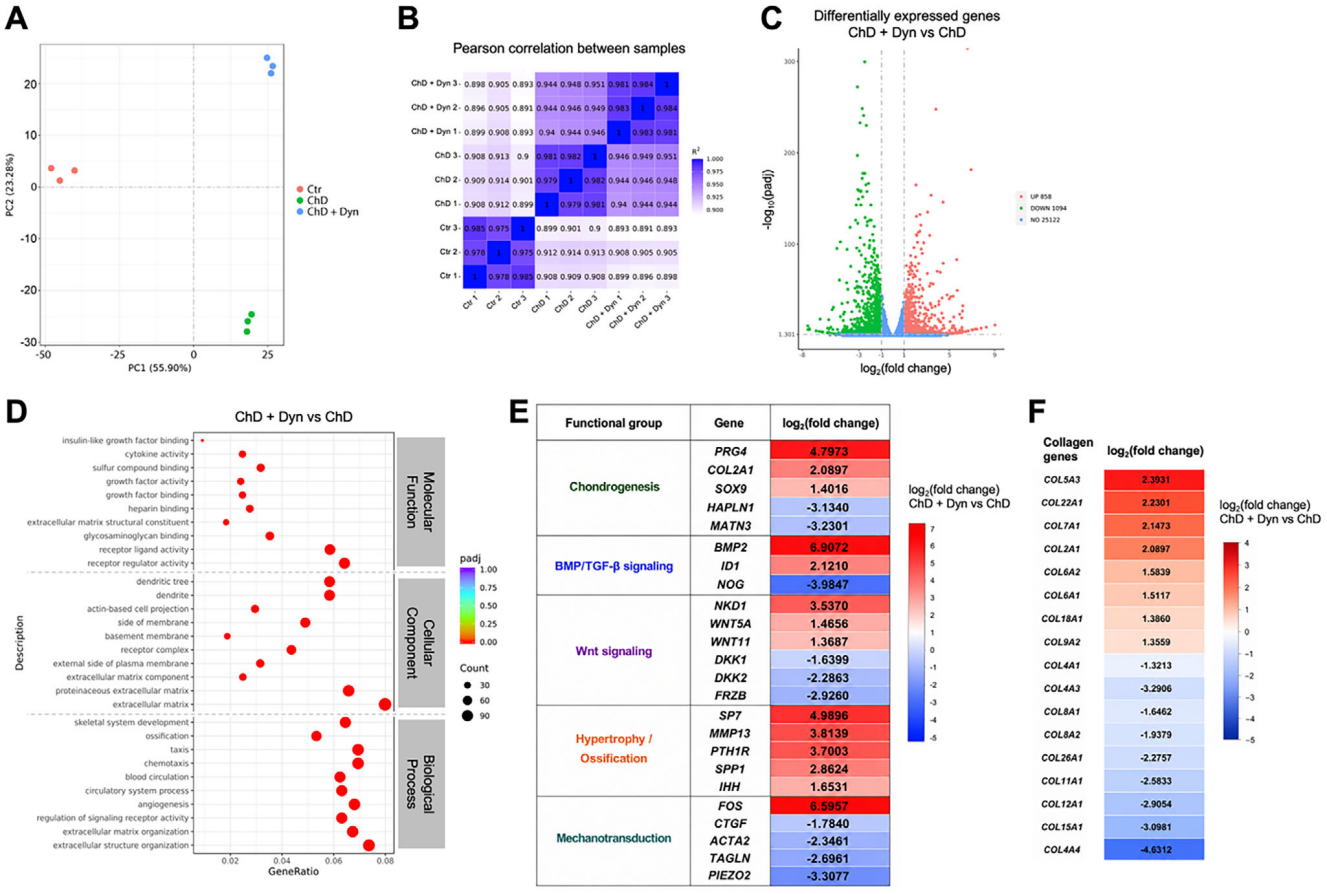
Transcriptomic analysis of MSCs cultured on PET scaffolds after 21 days of culture. (A) Principal component analysis (PCA) of normalized RNA‐sequencing data from control (Ctr), chondrogenic differentiation (ChD), and ChD combined with dynamic loading (ChD + Dyn) samples. (B) Pearson correlation heatmap of all replicates. (C) Volcano plot showing differentially expressed genes (DEGs) between ChD + Dyn and ChD. Red and green dots indicate significantly upregulated and downregulated genes, respectively, whereas blue dots represent genes that were not differentially expressed (|log_2_(fold change)| < 1 and/or adjusted *p* > 0.05). (D) Gene ontology (GO) enrichment analysis of DEGs identified in the ChD + Dyn versus ChD comparison. The top enriched terms are grouped into the three GO categories: molecular function, cellular component, and biological process. Dot size represents the number of genes associated with each GO term (gene count), whereas dot color corresponds to the adjusted *p*‐value (padj). The *x*‐axis indicates the gene ratio (the proportion of DEGs associated with each GO term). (E) Heatmap of representative DEGs in ChD + Dyn compared with ChD, grouped according to key biological pathways, including chondrogenesis, bone morphogenetic protein (BMP)/transforming growth factor‐β (TGF‐β) signaling, Wnt signaling, hypertrophy/ossification, and mechanotransduction. (F) Heatmap showing collagen DEGs in ChD + Dyn compared with ChD. Heatmap colors represent log_2_(fold change), with positive values, indicating upregulation and negative values indicating downregulation. For statistical analysis, a threshold of |log_2_(fold change)| ≥ 1.0 and an adjusted *p* value ≤ 0.05 were applied (*n* = 1 MSC donor; 3 independent experimental replicates).

Differential expression analysis of the ChD + Dyn versus ChD comparison identified 858 genes upregulated and 1 094 genes downregulated (adjusted *p* ≤ 0.05, |log_2_(fold change)| ≥ 1), as illustrated by the volcano plot (Figure 7C). GO enrichment analysis revealed significant enrichment of terms related to ECM organization, receptor‐mediated signaling, and developmental processes (Figure 7D). Enriched Molecular Function terms included receptor regulator activity and receptor ligand activity. Within the cellular component category, ECM and proteinaceous ECM were the most enriched terms. Biological process enrichment highlighted extracellular structure and ECM organization, but also angiogenesis and ossification.

To further characterize the molecular response to dynamic loading, representative DEGs associated with chondrogenesis, bone morphogenetic protein (BMP)/TGF‐β signaling, Wnt signaling, hypertrophy/ossification, and mechanotransduction were selected (Figure 7E). Compared with ChD alone, ChD + Dyn increased the expression of genes, including proteoglycan 4 (*PRG4*), *COL2A1*, *SOX9*, *BMP2*, Sp7 transcription factor (*SP7*), *MMP13*, and Fos proto‐oncogene (*FOS*), while downregulating genes such as noggin (*NOG*), matrilin 3 (*MATN3*), and piezo‐type mechanosensitive ion channel component 2 (*PIEZO2*). Analysis of collagen genes further demonstrated that dynamic loading altered ECM composition (Figure 7F). Although *COL2A1* and other collagen genes were upregulated, multiple fibrillar and matrix‐associated collagens, including *COL11A1*, *COL12A1*, and *COL15A1*, were downregulated.

### 3.6 Biomechanical Characterization of MSC‐Seeded PET Constructs

Scanning electron microscopy and micro‐computational tomography analysis confirmed a highly porous (85.15% ± 0.52% porosity), interconnected fibrous architecture with anisotropic fiber orientation and a pore size distribution predominantly between 0.01194 and 0.01989 mm, representing 54.8% of the total pore volume (Figure S6). Mechanical properties of the MSC‐seeded PET constructs were assessed by measuring permeability, equilibrium modulus, and aggregate modulus. The results were compared with baseline values obtained from non‐cultured, acellular PET scaffolds [33]. At 10% strain, the ChD constructs showed a moderate but significant reduction in permeability at Days 14 and 21 compared with Day 7 (*p* < 0.05; Figure 8A). However, no significant differences were found in equilibrium modulus or aggregate modulus between groups, time points, or strain levels (Figures 8B–C and Figure S7). Moreover, no significant differences were detected between the cultured constructs and the acellular controls (dashed line) for any of the measured biomechanical parameters.

**FIGURE 8:**
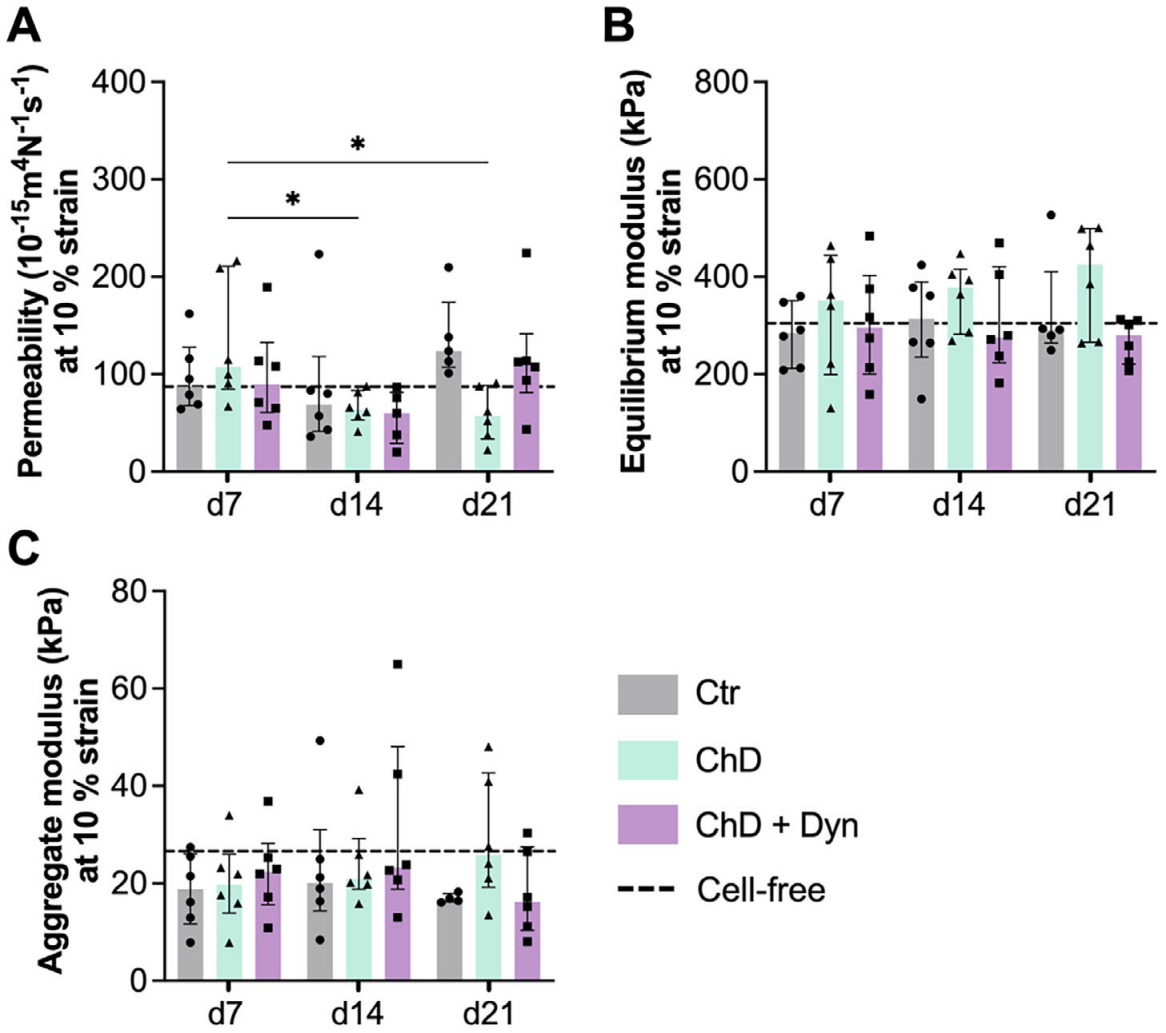
Biomechanical characterization of MSC‐seeded PET constructs at Days 7, 14, and 21 of culture under control conditions (Ctr), chondrogenic differentiation (ChD), and ChD combined with dynamic loading (ChD + Dyn). (A) Permeability (10^−15^m^4^N^−1^s^−1^), (B) equilibrium modulus (kPa), and (C) aggregate modulus (kPa) measured at 10% strain. Dashed lines indicate median values of biomechanical properties of non‐cultured cell‐free PET constructs. Data are presented as bar plots and as median with interquartile range (*n* = 1 MSC donor; 6 independent experimental replicates). * *p* < 0.05.

## 4 Discussion

This study evaluated the suitability of nonwoven PET scaffolds for meniscus tissue engineering and the influence of dynamic loading on the chondrogenic differentiation of human MSCs. MSCs from a single donor were intentionally used to minimize biological variability and enable a controlled assessment of the effects of chondrogenic differentiation and mechanical stimulation. The findings demonstrate that nonwoven PET scaffolds support MSC adhesion, viability, and differentiation while maintaining their mechanical stability throughout culture. Dynamic loading promoted MSC proliferation, transiently enhanced chondrogenic gene expression, and induced broad transcriptional changes involving ECM organization, mechanotransduction, and developmental signaling. Although dynamic loading did not increase overall ECM accumulation or improve construct biomechanics within 21 days, it altered ECM composition and modulated inflammatory and remodeling responses, indicating that its primary effect was to coordinate tissue remodeling rather than enhancing matrix synthesis.

Mechanical stimulation is of particular interest in meniscus tissue engineering because it mimics physiological joint loading, activates mechanotransduction pathways, and regulates cell behavior and matrix remodeling [51]. Szojka et al. demonstrated that hypoxic conditions combined with dynamic compression better mimic the physiological environment of the avascular inner meniscus and promote fibrocartilage‐related gene expression and matrix remodeling in engineered meniscus tissues [52]. Accordingly, hypoxia was combined with chondrogenic medium to establish conditions favorable for meniscus‐like tissue formation and to evaluate the additional effects of dynamic loading. The loading regime applied in this study (12% compressive strain, 1 Hz, 1 h/day), together with chondrogenic differentiation medium, was selected based on previous reports identifying these parameters as physiologically relevant for meniscus‐like tissue homeostasis [34, 35, 36].

Dynamic loading promoted MSC proliferation without increasing metabolic activity on a per‐cell basis, consistent with previous studies showing that physiological mechanical loading stimulates MSC expansion without compromising cell viability [1, 30, 34, 35, 36]. It also transiently enhanced the expression of the chondrogenic markers *SOX9*, *ACAN*, *COL1A1*, and *COL2A1*, while suppressing the hypertrophy marker *COL10A1* at Day 14. Similar responses have been reported in engineered cartilage and meniscus models, where cyclic compression accelerated early chondrogenic differentiation while limiting hypertrophic commitment [30, 31, 52, 53, 54]. Although these differences had largely converged by Day 21, RNA sequencing revealed persistent regulation of genes involved in ECM organization, mechanotransduction, and developmental signaling. Together, these findings suggest that dynamic loading primarily influenced the progression of MSC differentiation while inducing broader transcriptional changes associated with tissue remodeling.

Despite the transcriptional changes, dynamic loading did not increase ECM accumulation within the constructs. GAG content was significantly lower in ChD + Dyn versus ChD, whereas total collagen deposition remained comparable, resulting in lower GAG and collagen accumulation per cell. Rather than indicating impaired chondrogenesis, the lower GAG content likely reflects a combination of mechanical washout, altered proteoglycan retention, and dynamic regulation of ECM remodeling under cyclic compression [34, 55, 56, 57]. Consequently, preserved collagen deposition together with reduced GAG accumulation shifted the matrix composition toward a profile resembling that of the native meniscus [58]. Nevertheless, the newly synthesized collagen was likely immature and insufficiently organized to contribute to load‐bearing function, which may explain why these compositional changes were not accompanied by measurable improvements in the bulk mechanical properties of the constructs within the 21‐day culture period [59].

The mechanoadaptive response to dynamic loading was further reflected in the cellular secretory profile. The transient increase in PGE_2_ production in the ChD + Dyn group compared with ChD alone confirmed activation of mechanosensitive signaling without evidence of sustained inflammatory activation, consistent with the established role of the cyclooxygenase‐2 (COX‐2)/PGE_2_ axis in physiological responses to mechanical loading [53, 54, 55]. Dynamic loading also attenuated the secretion of several inflammatory mediators associated with OA progression, including IL‐6, IL‐12(p40), IL‐17A, eotaxin, GROα, IL‐8, and VEGF‐A, while partially restoring M‐CSF levels and maintaining PDGF‐AA production. IL‐6, IL‐17A, IL‐8, and GROα contribute to inflammatory signaling, matrix degradation, and inflammatory cell recruitment, whereas excessive VEGF‐A promotes pathological angiogenesis and hypertrophic cartilage remodeling [60, 61, 62]. Their reduced secretion suggests that dynamic loading shifted the cellular secretory profile toward a tissue‐remodeling phenotype. Consistent with this interpretation, gelatin zymography showed regulated MMP activity. Pro‐MMP‐2 increased with chondrogenic stimulation but was attenuated by the addition of dynamic loading, whereas MMP‐9 remained predominantly in its latent form, indicating controlled ECM turnover rather than progressive matrix breakdown [46].

Transcriptomic profiling provided mechanistic insight into the effects of dynamic loading. RNA sequencing revealed distinct transcriptional signatures between ChD + Dyn and ChD groups, with enrichment of biological processes related to ECM organization and extracellular structure organization, receptor‐mediated signaling, and developmental processes. These pathways are central to tissue remodeling and mechanotransduction and align with previous transcriptomic studies of mechanically stimulated engineered meniscus and fibrocartilaginous tissues [53, 63, 64]. ChD + Dyn induced selective ECM remodeling rather than a generalized increase in collagen synthesis compared to ChD alone. Upregulation of *COL2A1*, *COL9A2, PRG4*, *COL6A1*, and *COL6A2* suggests a meniscus‐like fibrocartilaginous phenotype and enhanced pericellular matrix remodeling [65, 66]. Conversely, reduced expression of *COL4A1*, *COL4A3*, *COL4A4*, *COL8A1*, *COL8A2*, *COL11A1*, *COL12A1*, and *COL15A1*, which contribute to basement membrane organization and collagen fibril assembly, further supports selective ECM remodeling rather than uniform matrix deposition [67]. Together, these findings indicate selective remodeling of ECM composition rather than generalized matrix synthesis. Dynamic loading also altered the expression of genes associated with TGF‐β, Wnt, and mechanotransduction signaling, which regulate fibrocartilage maturation and adaptation to mechanical loading and are also implicated in OA [68, 69, 70]. Notably, GO analysis identified enrichment of ossification‐related processes, with *SP7* and *MMP13* being upregulated after 21 days of culture. Although these genes are commonly associated with hypertrophic remodeling, they also participate in cartilage development and matrix turnover, and their expression alone is insufficient to conclude terminal hypertrophic differentiation [71, 72, 73]. This interpretation is supported by the transient suppression of *COL10A1* expression at Day 14, suggesting that hypertrophic signaling was temporally regulated rather than progressively sustained. Increased *COL10A1* and *MMP13* have been reported in engineered human meniscus constructs, where they were associated with meniscal degeneration and OA‐like remodeling [74]. In contrast, the concomitant upregulation of fibrocartilage‐associated ECM genes observed in the present study suggests that dynamic loading promotes adaptive matrix remodeling rather than a degenerative hypertrophic response. Nevertheless, the enrichment of ossification‐related biological processes suggests that prolonged mechanical stimulation may influence these developmental programs, warranting longer‐term studies to determine whether sustained loading further modulates their biological significance. As these findings are based on differential gene expression and pathway enrichment analyses, future studies integrating protein‐level analyses and functional validation will be required to confirm the mechanistic contribution of these pathways to mechanoadaptation.

No significant changes in equilibrium or aggregate moduli were detected during the 21‐day culture period. The biomechanical properties of the cell‐seeded constructs remained comparable to those of acellular PET scaffolds [33], indicating that the newly deposited ECM was insufficient to measurably alter the bulk mechanical behavior of the constructs. This is likely attributable to the intrinsic stiffness of the PET scaffold [75, 76], which masked the mechanical contribution of the newly synthesized matrix, together with the immature organization of the newly synthesized collagen [59]. Although GAGs are major contributors to compressive stiffness through their ability to retain water and generate osmotic swelling pressure [77, 78], the higher GAG accumulation observed under static chondrogenic conditions resulted only in a decrease in permeability over the 21‐days culture. This suggests that matrix deposition and maturation were insufficient to overcome the dominant mechanical contribution of the scaffold. Consequently, the mechanical properties of the constructs remained below those of native human meniscus, which possesses a highly organized collagen network and anisotropic mechanical properties [59]. Nevertheless, the mechanically stable and highly porous PET scaffold provided structural support while facilitating cell infiltration and matrix deposition. Because PET is a biostable, nondegradable polymer, long‐term functional performance of the construct will depend on effective tissue integration and remodeling within the persistent scaffold. These aspects cannot be assessed in the present in vitro study and will require long‐term in vivo evaluation. Accordingly, longer culture periods, post‐loading maturation phases, or more compliant composite scaffold designs may be required to translate the early biological responses observed here into measurable functional improvements [53, 56, 79].

Several limitations should be considered. First, a single loading protocol was investigated, representing a simplified approximation of the complex multiaxial loading experienced by the native meniscus, which is simultaneously subjected to compression, shear, and hoop stress [64]. Bioreactor systems combining dynamic compression, shear, and perfusion may better recapitulate physiological loading and further promote ECM maturation [80, 81]. Second, chondrogenic differentiation was performed under hypoxic conditions to mimic the avascular meniscal environment; therefore, the independent effects of hypoxia and chondrogenic supplementation could not be distinguished. Third, experiments were performed using MSCs from a single donor. Although no passage‐dependent differences were observed and the cells retained their differentiation potential, future studies including multiple donors are needed to determine the robustness and donor dependence of these responses. In addition, the 21‐day culture period was likely insufficient for ECM maturation. Previous studies have demonstrated that prolonged loading, or dynamic stimulation followed by static culture, can substantially increase collagen and GAG accumulation [56]. Lastly, the intrinsic stiffness of the PET scaffold may have masked the mechanical contribution of the newly synthesized matrix. Composite scaffolds that combine the mechanical stability of PET with a more cell‐permissive, ECM‐friendly phase, such as hydrogels, should be explored to accelerate maturation and unmask functional gains [55, 81].

Overall, dynamic compression of MSC‐seeded nonwoven PET scaffolds promoted cell proliferation, activated mechanosensitive signaling, and transiently regulated chondrogenic differentiation. Although dynamic loading did not increase overall ECM accumulation or improve construct biomechanics within 21 days, it is selectively altered ECM composition, modulated inflammatory and matrix remodeling responses, and induced transcriptional programs associated with mechanoadaptation. These results support the continued development of mechanically conditioned PET‐based constructs for meniscus tissue engineering and suggest that longer culture periods and optimized scaffold‐loading strategies will be required to translate these early biological responses into functional tissue maturation.

## 5 Conclusion

In summary, the present study demonstrates that nonwoven PET scaffolds provide a mechanically stable platform for human MSC‐based meniscus tissue engineering. Dynamic compression in combination with chondrogenic conditions promoted MSC proliferation, transiently enhanced chondrogenic differentiation, and induced broad mechanosensitive transcriptional responses involving ECM remodeling and developmental signaling. Although dynamic loading did not increase overall ECM accumulation or improve bulk mechanical properties within the 21‐day culture period, it altered ECM composition, resulting in lower GAG accumulation while maintaining collagen content, and regulated inflammatory and remodeling pathways. Collectively, these findings indicate that dynamic loading primarily modulates early MSC mechanoadaptive responses rather than simply enhancing matrix synthesis, highlighting the importance of mechanical stimulation during the early stages of meniscus tissue engineering.

## Author Contributions

**G.Q.T**.: conceptualization, methodology, investigation, formal analysis, data curation, visualization, and writing – original Draft. **L.d.R**.: investigation, data curation, and writing – review & editing. **A.L.F**.: investigation and writing – review & editing. **M.A**.: formal analysis, data curation, and writing – review & editing. **S.P**.: investigation, data curation, and writing – review & editing. **M.A**.: investigation and writing – review & editing. **A.I**.: supervision, resources, and writing – review & editing. **C.L**.: resources, methodology, and writing – review & editing. **A.M.S**.: conceptualization, methodology, supervision, project administration, funding acquisition, and writing – review & editing.

## Funding

Funded by the Deutsche Forschungsgemeinschaft (DFG, German Research Foundation)–Project number 465242756.

## Conflicts of Interest

The authors declare no conflicts of interest.

## Supporting information




**Supporting File 1**: adhm71574‐sup‐0001‐SuppMat.docx.

**Supporting File 2**: adhm71574‐sup‐0002‐SuppMat.docx.

**Supporting File 3**: adhm71574‐sup‐0003‐SuppMat.xlsx.

**Supporting File 4**: adhm71574‐sup‐0004‐SuppMat.xlsx.

**Supporting File 5**: adhm71574‐sup‐0005‐SuppMat.xlsx.

**Supporting File 6**: adhm71574‐sup‐0006‐SuppMat.xlsx.

## Data Availability

The data that support the findings of this study are available from the corresponding author upon reasonable request.
