# Supplementary material for: Dynamic Loading Regulates Meniscus‐Like Matrix Production in Human Mesenchymal Stromal Cell‐Seeded PET Scaffolds
Source: Adv Healthc Mater. 2026 Aug 11;15(39):e71574. doi: 10.1002/adhm.71574 (PMC13691407; doi:10.1002/adhm.71574)
Supplement: Supplementary file 1 [file ADHM-15-0-s001.docx]

Supporting Information

Dynamic Loading Regulates Meniscus-Like Matrix Production in Human Mesenchymal Stromal Cell-Seeded PET Scaffolds

Graciosa Quelhas Teixeira, Luisa de Roy, Anna-Lotta Feldmeier, Mubashir Ahmad, Sofia Pilão, Maria Ahrens, Anita Ignatius, Carsten Linti, and Andreas Martin Seitz*

Materials and Methods

Micro-computed tomography

Three-dimensional scaffold morphology was further characterized by Micro-computational tomography (μCT). Representative PET scaffolds were scanned using an isotrophic resolution of 3.98 μm, a source voltage of 40 kV and a current of 250 μA in a μCT scanning device (Skyscan 1172, Bruker microCT, Kontich, Belgium) and reconstructed to visualize the internal fiber network and evaluate structural homogeneity throughout the scaffold thickness. Cross-sectional images were generated in orthogonal planes to assess fiber distribution within the scaffold. Pore size distribution was determined from the reconstructed μCT datasets by quantifying the volume fraction of pores within predefined size ranges.

Histology

At each experimental time point, MSC-seeded PET constructs were fixed in 4% paraformaldehyde for 24 h. Safranin O/Fast Green staining was performed prior to cryosectioning. Briefly, constructs were rinsed in distilled water and stained with Weigert's iron hematoxylin (Waldeck, Münster, Germany), followed by 0.01% Fast Green (Waldeck) and 0.1% Safranin O (Sigma‐Aldrich, St. Louis, MO, USA), with brief differentiation in 1% hydrochloric acid and 1% acetic acid (VWR, Radnor, PA, USA) between steps. Following staining, samples were embedded in optimal cutting temperature compound (Leica Biosystems, Wetzlar, Germany), frozen at -20°C, and sectioned at a thickness of 10 µm using a cryostat (CM1950, Leica Biosystems). Sections were mounted on glass slides and imaged using a brightfield microscope (Axiophot, Zeiss, Jena, Germany). For quantitative analysis, three non-overlapping images were acquired at 100× magnification from the upper, central, and lower regions of each construct. The percentage of the extracellular matrix area was quantified using ImageJ (National Institutes of Health, Bethesda, MD, USA). A uniform threshold was applied to all images to identify positively stained regions, and the stained area was expressed as a percentage of the total construct area. The same thresholding and analysis parameters were used for all images. Measurements from the three images were averaged to obtain a single value for each region of each sample.

Figures

Figure S1. ****Cell distribution within PET scaffolds after MSC seeding.**** Representative scanning electron microscopy (SEM) images of cross-sections of MSC-seeded PET scaffolds acquired on day 1 after cell seeding. (A) Schematic representation of the regions evaluated by SEM: upper surface, central region, and lower surface of the scaffold. (B–D) Representative SEM images acquired on day 1 after cell seeding showing the upper surface (B), central region (C), and lower surface (D) of the PET scaffold. MSCs attached to PET fibers are indicated by red arrows, demonstrating cell presence throughout the scaffold thickness and supporting successful cell colonization beyond the scaffold surface. Scale bar = 200 μm.

**Figure S2.** **Representative scanning electron microscopy (SEM) images of MSC-seeded PET scaffolds cultured under control conditions (Ctr), chondrogenic differentiation conditions (ChD), and chondrogenic differentiation combined with dynamic loading (ChD + Dyn) after 7, 14, and 21 days of culture.** Images were acquired from the upper surface, central region, and lower surface of scaffold cross-sections at 100× (scale bar = 1 mm) and 500× (scale bar = 200 µm) magnification.

**Figure S3. Regional assessment of extracellular matrix deposition in MSC-seeded PET scaffolds cultured under control conditions (Ctr), chondrogenic differentiation conditions (ChD), and chondrogenic differentiation combined with dynamic loading (ChD + Dyn) after 7, 14, and 21 days of culture.** (A) Schematic representation of the regions analyzed within scaffold cross-sections, including the upper surface, central region, and lower surface. (B) Representative cross-sectional images of Safranin O/Fast Green-stained MSC-seeded PET scaffolds. Images were acquired from the upper surface, central region, and lower surface at 25× (scale bar = 1 mm) and 100× (scale bar = 200 µm) magnification. Safranin O stains sulfated glycosaminoglycans (red), whereas Fast Green counterstains collagen and other non-cartilaginous extracellular matrix components (green). (C) Quantification of matrix-positive area (%) in scaffold cross-sections at days 7, 14, and 21. Data are presented as bar plots and as median with interquartile range (n = 1 MSC donor; 3 independent experimental replicates). * *p* < 0.05.

**Figure S4.** **Extracellular matrix production by MSCs seeded on PET scaffolds at days 7, 14 and 21 of culture under control conditions (Ctr), chondrogenic differentiation (ChD), and ChD combined with dynamic loading (ChD + Dyn).** (A) GAG in supernatants (μg/mL). (B) Collagen in supernatants (μg/mL). Data are presented as bar plots and as median with interquartile range (n = 1 MSC donor; 8 independent experimental replicates). * *p* < 0.05, ** *p* < 0.01.

**Figure S5. Transcriptomic analysis of MSCs cultured on PET scaffolds after 21 days of culture under chondrogenic differentiation conditions (ChD) versus control (Ctr).** (A) Volcano plot showing differentially expressed genes between constructs cultured under ChD and Ctr. Red and green dots represent significantly upregulated and downregulated genes, respectively, whereas blue dots indicate genes that were not differentially expressed (|log_2_(fold change)| < 1 and/or adjusted *p* > 0.05). (B) Gene Ontology (GO) enrichment analysis of differentially expressed genes identified in the ChD versus Ctr comparison. The top enriched terms are grouped into the three GO categories: Molecular Function, Cellular Component, and Biological Process. Dot size represents the number of genes associated with each GO term (gene count), whereas dot color corresponds to the adjusted *p* value (padj). The x-axis indicates the gene ratio (the proportion of differentially expressed genes associated with each GO term). n = 1 donor; 3 independent experimental replicates.

Figure S6. ****Structural characterization of the non-woven PET scaffold.**** (A) Scanning electron microscopy (SEM) image showing the fibrous architecture of the non-woven PET scaffold scale bar = 200 μm). (B) Representative micro-computed tomography (μCT) reconstruction used for three-dimensional assessment of scaffold morphology and porosity. (C) Orthogonal μCT cross-sectional views demonstrating the internal structure and homogeneous fiber distribution throughout the scaffold thickness. (D) Pore size distribution analysis derived from μCT data, showing the volume fraction of pores within different size ranges.

**Figure S7.** **Biomechanical characterization of MSC-seeded PET constructs at days 7, 14 and 21 of culture under control conditions (Ctr), chondrogenic differentiation (ChD), and ChD combined with dynamic loading (ChD + Dyn).** (A) Permeability (10^-15^m^4^N^-1^s^-1^), (B) equilibrium modulus (kPa), and (C) aggregate modulus (kPa) measured at 15% and 20% strain. Dashed lines indicate median values of biomechanical properties of non-cultured cell-free PET constructs. Data are presented as bar plots and as median with interquartile range (n = 1 MSC donor; 5 – 6 independent experimental replicates).
