# Supplementary material for: Dynamic Loading Regulates Meniscus‐Like Matrix Production in Human Mesenchymal Stromal Cell‐Seeded PET Scaffolds
Source: Adv Healthc Mater. 2026 Aug 11;15(39):e71574. doi: 10.1002/adhm.71574 (PMC13691407; doi:10.1002/adhm.71574)
Supplement: Supplementary file 2 [file ADHM-15-0-s002.docx]

Supporting Information

Dynamic Loading Regulates Meniscus-Like Matrix Production in Human Mesenchymal Stromal Cell-Seeded PET Scaffolds

Graciosa Quelhas Teixeira, Luisa de Roy, Anna-Lotta Feldmeier, Mubashir Ahmad, Sofia Pilão, Maria Ahrens, Anita Ignatius, Carsten Linti, and Andreas Martin Seitz*

**Supporting Tables**

**Table S1.** Human oligonucleotide primers used for quantitative polymerase chain reaction. Primers were custom designed and purchased from Applied Biosystems. fw, forward; rev, reverse.

| Gene | Gene name | Sequence (forward and reverse primer) |
| --- | --- | --- |
| *ACAN* | aggrecan | fw: 5'-TCT GTA ACC CAG GCT CCA AC-3'  rev: 5'-CTG GCA AAA TCC CCA CTA AA-3' |
| *COL1A1* | collagen type I alpha 1 chain | fw: 5'- GAT TCC CTG GAC CTA AAG GTG C -3'  rev: 5'- AGC CTC TCC ATC TTT GCC AGC A -3' |
| *COL2A1* | collagen type II alpha 1 chain | fw: 5'-CGC ACC TGC AGA GAC CTG AA-3´  rev: 5'-TCT TCT TGG GAA CGT TTG CTG G-3' |
| *COL10A1* | collagen type X alpha 1 chain | fw: 5′-TCC TTG AAC TTG GTT CAT GGA GT-3′  rev: 5′-GGT GTT GGG TAG TGG TCC TT-3′ |
| *GAPDH* | glyceraldehyde-3-phosphate dehydrogenase | fw: 5‘- GAA GGT GAA GGT CGG AGT C-3’  rev: 5‘- GAA GAT GGT GAT GGG ATT TC-3’ |
| *SOX9* | SRY-box 9 | fw: 5'-AGT ACC CGC ACT TGC ACA AC-3'  rev: 5'-GCT TCT CGC TCT CGT TCA GA-3' |

**Table S2.** Differentially expressed genes associated with the Gene Ontology term "ossification" (GO:0001503) identified in the ChD + Dyn versus ChD comparison, including log₂(fold changes) and adjusted *p* values.

| Gene | Gene name | log_2_ (fold change) | Adjusted *p* |
| --- | --- | --- | --- |
| *BMP2* | bone morphogenetic protein 2 | 6.914473 | 2.1E-182 |
| *WNT7B* | Wnt family member 7B | 5.694372 | 3.41E-50 |
| *CYP24A1* | cytochrome P450 family 24 subfamily A member 1 | 5.620909 | 0.000987 |
| *TNFSF11* | TNF superfamily member 11 | 5.384497 | 3.57E-21 |
| *SP7* | Sp7 transcription factor | 4.993101 | 5.14E-41 |
| *MMP13* | matrix metallopeptidase 13 | 3.809858 | 1.5E-248 |
| *PTH1R* | parathyroid hormone 1 receptor | 3.702211 | 5.64E-23 |
| *CSGALNACT1* | chondroitin sulfate N-acetylgalactosaminyltransferase 1 | 3.563127 | 2.38E-57 |
| *FGFR3* | fibroblast growth factor receptor 3 | 3.56234 | 6.94E-09 |
| *SPP1* | secreted phosphoprotein 1 | 2.861146 | 1.09E-10 |
| *S1PR1* | sphingosine-1-phosphate receptor 1 | 2.813236 | 1.35E-13 |
| *OSR1* | odd-skipped related transciption factor 1 | 2.768704 | 2.73E-11 |
| *MGP* | matrix Gla protein | 2.622432 | 9.55E-53 |
| *LRP4* | LDL receptor related protein 4 | 2.268663 | 1.22E-22 |
| *AREG* | amphiregulin | 2.162496 | 1.3E-05 |
| *SNAI1* | snail family transcriptional repressor 1 | 2.129389 | 8.26E-43 |
| *ID1* | inhibitor of DNA binding 1. HLH protein | 2.122131 | 2.1E-110 |
| *COL2A1* | collagen type II alpha 1 chain | 2.089724 | 0.043928 |
| *PENK* | proenkephalin | 1.764425 | 1.19E-36 |
| *IHH* | indian hedgehog | 1.647996 | 0.003744 |
| *FASN* | fatty acid synthase | 1.580846 | 5.57E-45 |
| *JUNB* | JunB proto-oncogene. AP-1 transcription factor subunit | 1.558361 | 4.23E-50 |
| *COL6A1* | collagen type VI alpha 1 chain | 1.511724 | 1.19E-37 |
| *WNT5A* | Wnt family member 5A | 1.468426 | 1.84E-67 |
| *SOX9* | SRY-box 9 | 1.396374 | 2.7E-25 |
| *WNT11* | Wnt family member 11 | 1.365391 | 1.5E-15 |
| *TWIST2* | twist family bHLH transcription factor 2 | 1.215202 | 1.33E-09 |
| *IFT80* | intraflagellar transport 80 | 1.197023 | 2.21E-15 |
| *SMAD6* | SMAD family member 6 | 1.190131 | 3.81E-12 |
| *SMOC1* | SPARC related modular calcium binding 1 | 1.136301 | 5E-18 |
| *TGFB3* | transforming growth factor beta 3 | 1.064868 | 2.56E-26 |
| *FOXC1* | forkhead box C1 | 1.044426 | 7.29E-12 |
| *CSF1* | colony stimulating factor 1 | 1.04066 | 1.11E-16 |
| *PHEX* | phosphate regulating endopeptidase homolog X-linked | 1.040473 | 0.031043 |
| *KREMEN1* | kringle containing transmembrane protein 1 | 1.028648 | 2.29E-11 |
| *FHL2* | four and a half LIM domains 2 | -1.02251 | 2.53E-31 |
| *CYR61* | cysteine rich angiogenic inducer 61 | -1.04533 | 3.79E-30 |
| *PKDCC* | protein kinase domain containing, cytoplasmic | -1.06151 | 0.003888 |
| *CDH11* | cadherin 11 | -1.14002 | 2.96E-53 |
| *IL6ST* | interleukin 6 signal transducer | -1.15055 | 2.3E-35 |
| *PTHLH* | parathyroid hormone like hormone | -1.15732 | 5E-08 |
| *CHRD* | chordin | -1.17083 | 4.12E-06 |
| *SMAD3* | SMAD family member 3 | -1.18018 | 1.81E-24 |
| *FSTL3* | follistatin like 3 | -1.23617 | 2.55E-57 |
| *BMP6* | bone morphogenetic protein 6 | -1.24296 | 0.007512 |
| *RBPJ* | recombination signal binding protein for immunoglobulin kappa J region | -1.24783 | 1.89E-44 |
| *BMPR2* | bone morphogenetic protein receptor type 2 | -1.25993 | 5.66E-45 |
| *IGFBP3* | insulin like growth factor binding protein 3 | -1.26129 | 1.82E-06 |
| *LRP5* | LDL receptor related protein 5 | -1.26428 | 1.99E-28 |
| *RANBP3L* | RAN binding protein 3 like | -1.40474 | 2.42E-06 |
| *VCAN* | versican | -1.4353 | 2.83E-60 |
| *SEMA7A* | semaphorin 7A | -1.44066 | 5.06E-58 |
| *TUFT1* | tuftelin 1 | -1.5309 | 5.67E-19 |
| *DKK1* | dickkopf WNT signaling pathway inhibitor 1 | -1.64345 | 0.0001 |
| *PHOSPHO1* | phosphoethanolamine/phosphocholine phosphatase | -1.67264 | 4.41E-05 |
| *ITGA11* | integrin subunit alpha 11 | -1.68079 | 9.66E-12 |
| *SFRP1* | secreted frizzled related protein 1 | -1.72227 | 0.000956 |
| *NPNT* | nephronectin | -1.7356 | 0.035728 |
| *TEK* | TEK receptor tyrosine kinase | -1.75823 | 3.54E-60 |
| *CTGF* | connective tissue growth factor | -1.78256 | 2.2E-111 |
| *FBN2* | fibrillin 2 | -1.79047 | 3.74E-31 |
| *INTU* | inturned planar cell polarity protein | -1.83058 | 1.1E-35 |
| *PTGER4* | prostaglandin E receptor 4 | -1.97519 | 9.48E-12 |
| *SORT1* | sortilin 1 | -2.05261 | 4.4E-127 |
| *STC1* | stanniocalcin 1 | -2.0685 | 8.23E-24 |
| *FGFR2* | fibroblast growth factor receptor 2 | -2.47587 | 1.44E-67 |
| *DHRS3* | dehydrogenase/reductase 3 | -2.49768 | 5.57E-09 |
| *RASSF2* | Ras association domain family member 2 | -2.49877 | 1.32E-25 |
| *COL11A1* | collagen type XI alpha 1 chain | -2.58331 | 1.5E-241 |
| *IBSP* | integrin binding sialoprotein | -2.58374 | 1.06E-05 |
| *SLC8A1* | solute carrier family 8 member A1 | -2.60637 | 3.08E-46 |
| *ACVR2A* | activin A receptor type 2A | -3.02167 | 1.6E-159 |
| *GDPD2* | glycerophosphodiester phosphodiesterase domain containing 2 | -3.53543 | 0.049898 |
| *IL6* | interleukin 6 | -3.69927 | 8.16E-05 |
| *NOG* | noggin | -3.97875 | 2.19E-51 |
| *LEP* | leptin | -4.25626 | 4.64E-11 |
